# Solid-state characterization of triamcinolone acetonide nanosuspensiones by X-ray spectroscopy, ATR Fourier transforms infrared spectroscopy and differential scanning calorimetry analysis

**DOI:** 10.1016/j.dib.2017.09.002

**Published:** 2017-09-13

**Authors:** Eva García-Millán, Mónica Quintáns-Carballo, Francisco Javier Otero-Espinar

**Affiliations:** a Departamento de Farmacología, Farmacia y Tecnología Farmacéutica, Facultad de Farmacia, Universidad de Santiago de Compostela, Campus Vida s/n, 15782 Santiago de Compostela, Spain; b Instituto de Farmacia Industrial, Facultad de Farmacia, Universidad de Santiago de Compostela, Campus Vida s/n, 15782 Santiago de Compostela, Spain

**Keywords:** Triamcinolona acetonide nanosuspensiones, X-ray spectroscopy, FTIR spectroscopy, DSC

## Abstract

The data presented in this article describe the physical state of the triamcinolone acetonide (TA) in nanosuspension stabilized with polyvinyl alcohol (PVA) and poloxamer 407 (PL). The data were assessed by X-ray spectroscopy, ATR Fourier transforms infrared spectroscopy measurements (FTIR), and Differential scanning calorimetry (DSC) analysis. PVA, PL and polymeric mixture (PVA and PL) were compared with nanosuspension and the interactions between drug triamcinolone acetonide and polymers were studied. The data are related and are complementary to the research article entitle “Improved release of triamcinolone acetonide from medicated soft contact lenses loaded with drug nanosuspensions” (García-Millán et al., 2017) [1].

**Specifications Table**

**Table:** 

Subject area	*Pharmaceutical Technology*
More specific subject area	Solid state characterization by X-ray spectroscopy, ATR Fourier transforms infrared spectroscopy, differential scanning calorimetry.
Type of data	*X-ray, ATR Fourier transforms infrared spectroscopy and DSC graphs, text*
How data was acquired	*X-ray Philips diffractometer; Varian FTIR 670 and DSC Q-100 apparatus*
Data format	*Analyzed*
Experimental factors	In order to remove water of nanosuspensions the samples were frozen with liquid N_2_ and were freeze-dried (A Telstar LyoQuest Plus)
Data source location	*Santiago de Compostela, Spain*
Data accessibility	*The data are with this article*

**Value of the data**

• This data will be helpful for the research community that characterizes the solid state of the drug in nanosuspensions used for loading media in soft contact lenses, stabilized with PVA and PL.
• This data allows the scientific community to know the solid state of triamcinolone acetonide, selected triamcinolone acetonide nanosuspension, polymers PVA and PL used as stabilizers and the polymeric mixture using X-ray, FTIR and DSC techniques.

## 1 Data

Stable triamcinolone acetonide nanosuspensions have been designed and synthesized by nanoprecipitation technique to formulate TA in high concentrations. The nanosuspension was selected in order to use it as loading media and to obtain medicated soft contact lenses. Nanosuspension selected was FM2, a monodisperse nanosuspensions in nanometric range, with a mean particle size of 147 nm and a TA load of 0.8 mg/ml, using mixtures of PVA (10%) and PL (10%) as stabilizers prepared by the nanoprecipitation technique [1]. Nanometric size of the TA particles can promote an increase of the solubility of the drug in nanosuspensions. The solid-state characterization nanosuspension can help to explain this behavior. Also, physical state of drug and polymers may have an important influence on the drug loading process and *in vitro* release characteristics.

**Fig. 1:**
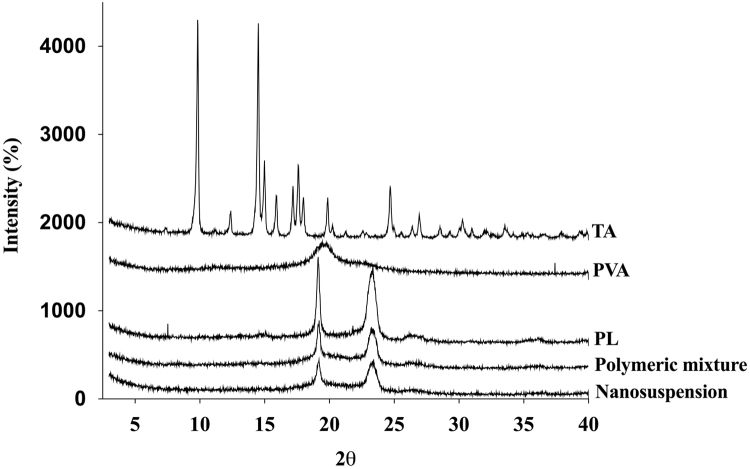
X-ray diffraction patterns of triamcinolone acetonide, polymers and freeze-dry nanosuspension.

**Fig. 2:**
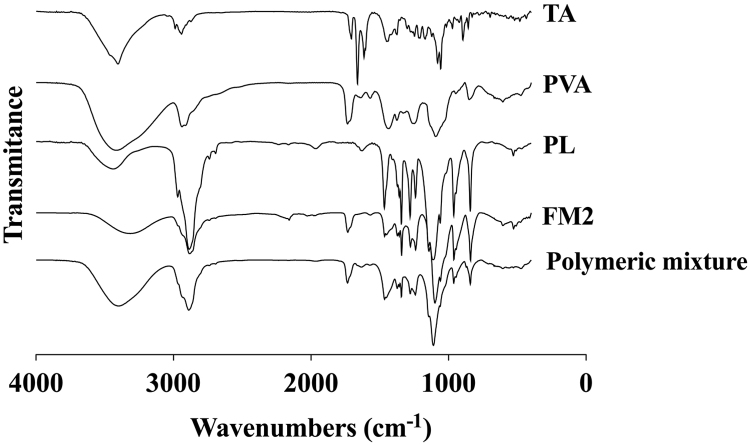
FTIR spectra of the triamcinolone acetonide, polymers and freeze-dry nanosuspension.

**Fig. 3:**
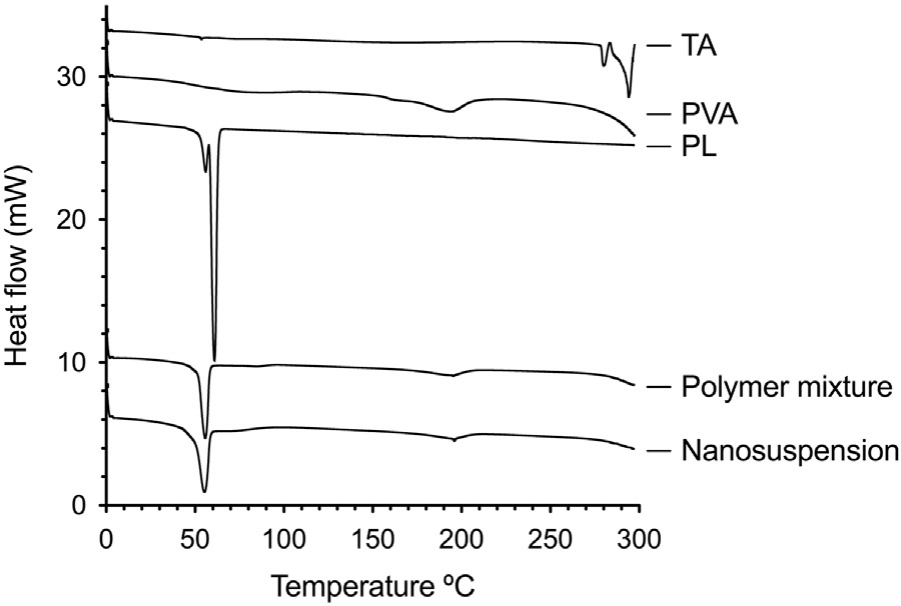
DSC traces of triamcinolone acetonide, polymers and freeze-dry nanosuspension.

The dataset of this article shows additional information about the solid state of the drug and polymers used to elaborate the nanosuspension. The Fig. 1, Fig. 2, Fig. 3 show the physical state of samples assessed by X-ray spectroscopy, FTIR and DSC analysis.

Fig. 1 shows X-ray diffraction patterns of the drug, polymers and freeze-dry nanosuspension prepared by nanoprecipitation as describes in García-Millán et al. [1]. Intact TA X-ray diffraction data shows sharp peaks in the range 2*θ*=9–21° and 24–28°, specifically at 2*θ*=9°, 14°, 17° and 24°, which is indicative of the typical crystal structure of the drug [2], [3]. Data of a sample of pure PVA shows some diffraction bands with a broad peak at 2*θ*=19.5° hence is identified as a semicrystalline structure [2], [4]. The PL X-ray pattern data shows two characteristic peaks with the highest intensity at diffraction angles at 2*θ*=18.3° and 22.8° belong to crystalline Poloxamer block [5], [6]. X-ray diffraction data results from nanosuspension show two peaks of less intensity at 2*θ*=18° and 22° corresponding to the polymers. Nanosuspension X-ray pattern data do not show the characteristic peaks of the drug, indicating that in this system the TA is in the amorphous state.

Fig. 2 shows the ATR-FTIR infrared spectra. Pure TA spectra data shows a typical infrared absorption bands at 3392 cm^−^^1^ that can be associated with the stretching vibration of hydrogen bonded hydroxyl, and at 1726 cm^−^^1^, corresponding to stretching vibration of the carbonyl group at aliphatic ester bonds. Other infrared absorption bands observed are typical of triamcinolone acetonide at 1122 cm^−1^ – asymmetric axial deformation of C–O–C bond in aliphatic esters and at 1055 cm^−^^1^ – stretching vibration of C-F [7].

In PVA FTIR data, all major peaks related to hydroxyl and acetate groups were observed. The large bands observed between 3550 and 3200 cm^−1^ are linked to O-H stretching vibration from the intermolecular and intramolecular hydrogen bonds. The vibrational band observed between 2840 and 3000 cm^−1^ refers to C-H stretching vibration from alkyl groups and the peaks between 1750 and 1735 cm^−^^1^ are due to the stretching C=O and C–O from acetate group remaining from PVA [8].

FTIR data of pure PL is characterized by principal absorption peaks at 2889 cm^-1^ (C-H stretching vibrations), 1342 cm^−^^1^ (O-H bend) and 1111 cm^−^^1^ (C-O-C stretching vibrations) [5].

FTIR absorption bands of pure drug and polymers were also observed on the nanosuspension spectra data. The difference among curves is slight, but it is noted a reduction of the intensity in the sharpness of the O-H peaks between 3550 and 3200 cm^−^^1^ of FM2. Several infrared absorption bands of PL are clearly observable in the FTIR spectrum of nanosuspension, but with less intensity.

FTIR spectrum data of polymeric mixture shows more intensity in the peaks corresponding to the polymers; around 3000 cm^−^^1^ for PVA and PL, between 1750 and 1735 cm^−^^1^ for PVA and between 1000 and 1300 cm^−^^1^ for PL.

Fig. 3 shows the DSC curves for pure TA, polymers PVA and PL, nanosuspension and polymeric mixture. According to DSC curve, TA has an endothermic band at 292 °C, which corresponds with the melting point of the TA in crystalline anhydrous state [3]. For pure polymers, PL presents a double peak between 52 and 57 °C which belongs to the melting crystalline [6], [9], [10] and PVA has a melting peak temperature (T_m_) of 202.7 °C [11] corresponding to the melting of the crystalline fraction of the polymer and an endothermic event that start up to 280 °C corresponding to the beginning of the glass transition temperature band of the amorphous phase [12].

The DSC data of nanosuspension and the polymeric mixture shows a combination of the thermal events of PL and PVA. These DSC traces showed a peak of lesser intensity than the PL at about 60 °C and another peak at about 200 °C and the beginning of an endothermic event up to 280 °C corresponding to PVA. In the trace of nanosuspensions, the melting event of the drug in 292 °C disappears, indicating that drug is in the amorphous state.

## 2 Experimental design, materials and methods

### 2.1 Materials

The drug TA was supplied by Roig Farma (Spain), poloxamer 407 (PL, from 9840 to 14.600 Da) and polyvinyl alcohol (PVA; 87–90% hydrolysed, 30.000–70.000 Da), were purchased from Sigma-Aldrich Chemical (Spain).

### 2.2 Preparation of samples: freeze-drying nanosuspension

The nanosuspensions were prepared by nanoprecipitation technique according [1]. PL and PVA 10% (w/v) in water were used as an aqueous phase. Drug solutions 2 mg/ml were prepared in acetone. The organic phase was slowly added to the aqueous solution at room temperature under stirring at 13.500 rpm (high speed homogenizer Ultra Turrax T25 Janke & Kunkel, IKA1, Germany). The acetone was removed by evaporation at 85 °C in a rotary evaporator (Labo Rota 300, Resona Techins, Switzerland with a Labo Therm SW 200 water-bath) to a final volume of 5 ml.

To remove water, the samples were frozen with liquid N_2_ and were lyophilized using a freeze-drying system (A Telstar LyoQuest Plus, equipped with a vacuum pump system Telstar 2G-6, Spain) at 0.005 mbar and at −79 °C.

### 2.3 X-ray spectroscopy

The crystalline powder diffraction measurements were made by Philips diffractometer type, handled with a unit control type “PW1710”, a vertical goniometer type “PW1820/00” and a generator type “Enraf Nonius FR590“ operating 40 kV and 30 mA. X-rays were obtained from a sealed Cu tube and radiation was monochromatic with graphite monochromator (λKα1=1.5406 Å). The XRD diffractogram patterns were obtained in the angular range of 2<2*⊖*<40 with a step size of 0.02° and a measuring time of 2 s per step. The samples were turned over to obtain profiles optimal peak for analysis, as well as minimize the effect of preferential orientation. They were deposited on a basis of a crystal oriented to avoid background noise (scattering) caused by a vitreous support.

### 2.4 ATR Fourier transforms infrared spectroscopy

The ATR-FTIR spectra were obtained using a spectrometer Varian FTIR 670 (Varian Inc., USA) with a resolution of 0.10 cm^−1^.

### 2.5 Differential scanning calorimetry analysis

DSC curves were performed using a DSC Q-100 apparatus (TA Instruments, UK) equipped with a refrigerated cooling accessory. The samples were weighted in an aluminium pan and then heated from 30 to 100 °C, cooled to 0 °C and heated again up to 300 °C, at rate of 10 °C/min. Nitrogen was used as purge gas at a flow rate of 50 ml/min. The calorimeter was calibrated for constant cell and temperature using indium (melting point 156.61 °C, enthalpy of fusion 28.71 J/g), and for constant heat capacity using sapphire standards.
